# Robot-Assisted Removal of a Broken Scalpel Blade following Discectomy

**DOI:** 10.1155/2019/8609246

**Published:** 2019-06-04

**Authors:** Christos Koutserimpas, Argyrios Ioannidis, Michael Konstantinidis, Panagiotis Athanasopoulos, Fotios Antonakopoulos, Konstantinos Konstantinidis

**Affiliations:** ^1^Department of Orthopaedics and Traumatology, “251” Hellenic Air Force General Hospital of Athens, Greece; ^2^Department of General, Bariatric, Laparoscopic and Robotic Surgery, Athens Medical Center, Athens, Greece

## Abstract

The risk of a broken scalpel blade during discectomy is considered extremely rare, while no guidelines exist regarding this complication. We report a case of a robotic broken blade removal following lumbar discectomy. A 52-year-old female was subjected to L4-L5 discectomy. During the annulus resection, the scalpel blade broke and was retained within the disc space. The broken blade migrated towards the abdominal cavity and viscera. Emergency CT angiography scan revealed that the main vessels were intact, while the broken surgical knife was located anterior to the lumbar spine at the L4/L5 level, to the left of the aorta and superiorly of the left common iliac artery. At that point, robot-assisted laparoscopy was performed. The broken instrument was located and carefully removed. It seems more proper that such foreign bodies should be removed, while robotic surgery may play a significant role in cases that the foreign body is near major vessels.

## 1. Introduction

Robotic, computer-assisted, or robot-assisted surgery are similar terms for the use of robotic systems aiding in various surgical procedures. Robotic surgery has been developed in order to overcome limitations of minimally invasive surgery and to increase the capabilities of open surgery [1, 2].

The main advantages of computer-assisted surgery for the surgeon are as follows: greater visualization, enhanced dexterity in which dissections can be performed, and greater precision. Since its introduction, robotic surgery rapidly expanded in a plethora of surgical subspecialties, as well as operations. It has been estimated that over 3 million patients have been operated since the early 2000s with the introduction of the da Vinci® device [1].

There have been a few cases reported in the literature so far, describing the use of robotic surgery for iatrogenic complications, such as foreign body removals and the cement removal following percutaneous vertebroplasty [3]. The risk of a broken scalpel blade during discectomy is considered extremely rare, while no guidelines exist regarding this complication, since such cases are rarely published due to medicolegal implications [4]. The robot-assisted removal of such iatrogenic foreign bodies has not been described so far.

A case of a foreign body removal (a broken no. 11 scalpel blade), during a lumbar discectomy, with the use of the “da Vinci® Robotic System” is presented, exhibiting the advantages of computer-assisted surgery for the surgeon.

## 2. Case Presentation

**Figure 1:**
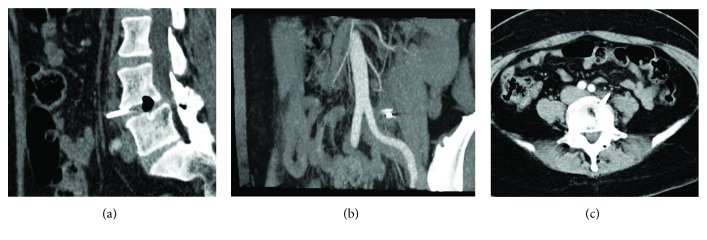
(a) Sagittal, (b) coronal, and (c) axial computer tomography angiography views, revealing the broken no. 11^th^ scalpel blade in front of the L4/L5 intervertebral space.

A 52-year-old female, with an unremarkable medical history, with the exception of lumbar herniation, was subjected, under general anesthesia and in a genupectoral position, to L4-L5 discectomy. During the annulus resection, the no. 11 scalpel blade broke and it was retained within the disc space. Attempts to remove the foreign body were performed under fluoroscopy. However, the broken blade migrated towards the abdominal cavity and viscera. Immediately, a CT angiography scan was performed, in order to locate the broken instrument (Figure 1). CT angiography revealed that the main vessels were intact, while the broken surgical knife was located anterior to the lumbar spine at the L4/L5 level and to the left of the aorta.

**Figure 2:**
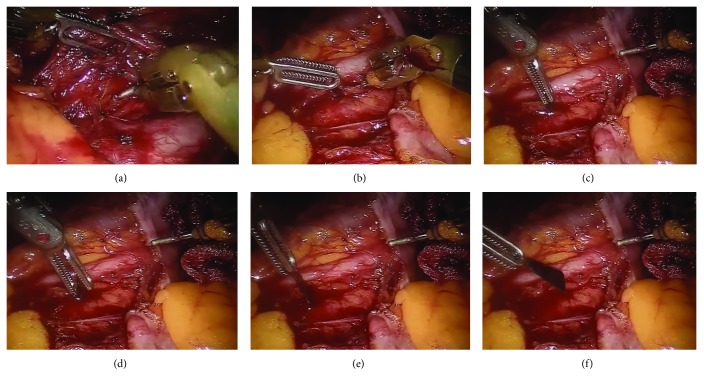
Intraoperative pictures of the robot-assisted removal of the broken blade.

The patient remained during this procedure stable, at all times. Urgently, the patient was placed in a supine position and a robot-assisted laparoscopy was initiated. Under general endotracheal anesthesia, the da Vinci® platform was brought to the operating table between the patient's legs. The camera port was inserted inferior and to the right of the umbilicus using the Hasson technique. Under direct vision, two additional robotic arm trocars were inserted at the right and left iliac fossa, respectively. Once the robot was docked, an exploration of the peritoneal cavity was performed. With the patient tilted to the right, the small intestine was transferred to the right abdominal cavity so that the retroperitoneum below the level of the left kidney could be exposed. The retroperitoneum was carefully dissected using bipolar Cadier forceps on the left arm and monopolar scissor and hook on the second arm. After the access to the abdominal aorta was gained, the broken scalpel was identified in close distance to the aorta and the left common iliac artery with no signs of active bleeding. The scalpel was slowly removed with extreme care not to traumatize the vessels (Figure 2). A final abdominal exploration was performed before the platform was removed (Video 1). The patient was deintubated without complications and was transferred to the recovery room.

The patient had an uneventful hospitalization and was discharged at the 3^rd^ postoperative day. At follow-up, 2 years after the operation, she remains without any signs or symptoms of disease.

## 3. Discussion

Breaking of the surgical scalpel blade during lumbar discectomy has already been described as associated with the procedure risk [4]. Lumbar discectomy has become a common operation, and its incidence is increasing. Such iatrogenic complications may have a higher incidence than the one reported so far in the literature, since they are rarely published. Medicolegal implications prohibit surgeons from reporting such cases [5]. Therefore, there is a lack of data regarding this complication. Literature is scarce, and no clear guidelines exist regarding the management of such cases. However, the present case is the first description in the literature of a robot-assisted removal of such an object.

Amirjamshidi et al. in 1994 was the first one to report 4 cases of a broken scalpel blade within the intervertebral disc space and migration towards the abdominal cavity and viscera [6]. The authors concluded that the no. 15 surgical knife was more prone to break during the cutting of firm and calcified annulus and posterior longitudinal ligament [6]. Attempts to remove the broken instrument were encouraged in cases that the surgeon was able to see the broken fragment within the disc space. However, such attempts may cause the further descent of the foreign body in deep disc space. In such cases, fluoroscopy was necessary. Therefore, when this is not available, the patient should be kept in close monitoring and immediately referred to a center with those facilities [6].

Complications due to broken blades and spontaneous migration have already been reported by De Praetere et al. [7], while a broken sharp scalpel left in an intervertebral space and slipped to pelvic cavity was also reported by Li et al. [8].

This may lead to serious complications, depending upon the location of the broken scalpel blade. The risk of intra-abdominal visceral and vascular injuries is high [6–8]. In the reported case, the broken scalpel blade migrated anterior of the L4/L5 disc space just about 1 cm left of the abdominal aorta, which could lead to extremely serious complications. The decision to approach the foreign object through the abdomen was made due to its location. Considering the enhanced dexterity which robotic surgery offers when compared to laparoscopic techniques and the great experience of our center, the decision to perform the removal robotically assisted was made.

Due to lack of data, there are not clear guidelines regarding this iatrogenic complication. There are still many issues that need to be clarified, such as whether conservative treatment is an option for asymptomatic foreign bodies without associated risks and whether it is better to remove the foreign body in a second intervention and not during the initial operation [6, 7]. It seems more proper that due to the high risk of visceral injuries, such objects should be removed.

Furthermore, other options for removal of the broken scalpel should be mentioned. In the present case, the part of the broken blade remained in the intervertebral disc space; therefore, if the operating room was equipped with intraoperative CT, CT-guided removal of the broken scalpel blade through disc space could be a feasible option. The main advantage of this approach would be the avoidance of repositioning the patient from prone to supine positions which could lead to surrounding tissue injuries. Additionally, an issue that should be considered is whether insufflation of the abdominal cavity may have risk for injury of tissues surrounding the broken blade. Hence, open procedure should always be kept in mind.

Robotic surgery is an advanced form of minimally invasive or laparoscopic (small incision) surgery where surgeons use a computer-controlled robot to assist them in certain surgical procedures [9, 10]. The robot's “hands” have a high degree of dexterity, allowing surgeons the ability to operate in very tight spaces in the body that would otherwise only be accessible through open (long incision) surgery. Robotic assisted surgery has offered some major advantages for surgeons as well, such as greater visualization, enhanced dexterity, and greater precision [1, 2, 9–11].

The main advantages of computer-assisted surgery over classical surgery regarding the patient are decreased blood loss, smaller incisions, less pain and hospital stay, and quicker healing, while the main criticism regarding this type of surgery is the increased cost per operation [3, 4], whereas the main disadvantages are the high cost ($1.25 million for the da Vinci® Robotic System as of 2004 [3]), the size of the system, prohibiting its use in small operating rooms, and the longer operative times when compared to similar laparoscopic approaches [4].

## 4. Conclusion

The presented case is the first case reporting the robot-assisted removal of a broken blade following discectomy. The high-quality visualization and the enhanced dexterity were of utmost importance for the removal of the potentially life-threatening foreign body. Such cases should be reported, since no clear guidelines exist for their management. It seems more proper that broken blades should be removed. If the broken scalpel blade is near major vessels or in difficult-to-approach regions, the use of robot-assisted surgery by experienced surgeons could be an option.

## Supplementary Materials

Supplementary Materials Video 1: robotic assisted removal of the broken blade.
